# Mesenchymal intravenous stromal cell infusions in children with recessive dystrophic epidermolysis bullosa: MissionEB protocol for a randomised, double-blinded, placebo-controlled, two-centre, crossover trial with an internal phase I dose de-escalation phase and open-label extension

**DOI:** 10.1136/bmjopen-2024-089857

**Published:** 2025-05-21

**Authors:** Maria L Bageta, Pablo López-Balboa, Munyaradzi Dimairo, Rachel Glover, Kate Hutchence, Diana Papaioannou, Cindy Cooper, Katie Biggs, Paul Tappenden, Katherine Ennis, Malobi Ogboli, Marie-Louise Lovgren, Pratima Poudel, Muna Nadeem, John A McGrath, Steven A Julious, Gabriela Petrof, Anna E Martinez

**Affiliations:** 1 Department of Dermatology, Great Ormond Street Hospital for Children NHS Foundation Trust, London, UK; 2 Clinical Trials Research Unit, ScHARR, The University of Sheffield, Sheffield, UK; 3 School of Health and Related Research, The University of Sheffield, Sheffield, UK; 4 Department of Dermatology, Birmingham Children’s Hospital NHS Foundation Trust, Birmingham, UK; 5 King’s College London, London, UK

**Keywords:** DERMATOLOGY, Paediatric dermatology, Clinical Trial, Mesenchymal Stem Cells

## Abstract

**Introduction:**

Recessive dystrophic epidermolysis bullosa (RDEB) is a severe genetic mucocutaneous fragility disorder characterised by chronic blistering, slow wound healing and increased risk of squamous cell carcinoma. Current management options are very limited.

**Methods:**

This is a randomised (1:1), placebo-controlled, double-blinded crossover (A/B) trial with an internal phase I dose de-escalation (4+5 design) in the first 3 months and a 12-month continued treatment follow-on open-label study if 3-month outcome data from the crossover trial indicate safe and beneficial effects. RDEB is a rare condition, so we expect to recruit a maximum of 36 participants based on feasibility and not formal power considerations. Participants aged>6 months and <16 years will be recruited at Great Ormond Street Hospital and Birmingham Children’s Hospital. They will receive 2–3×10^6^ cells/kg intravenous infusion of umbilical cord-derived mesenchymal stem cells or placebo at the start of each crossover period (day 0) and 14 days later. The dose will be de-escalated to 1–1.5×10^6^ cells/kg depending on observed toxicity. For the main crossover trial, the primary outcome is the change in disease severity as measured by the Epidermolysis Bullosa Disease Activity and Scarring Index at 3 months from day 0 infusion. Secondary outcomes measured at 3 and 6 months from day 0 infusion include changes in general clinical appearance of skin disease, pain and itch, and quality of life. Adverse events and serious adverse events will be monitored throughout the trial.

**Ethics and dissemination:**

North East—York Research Ethics Committee approved the protocol (ref: 21/NE/0016) on 16 March 2021. Findings will be published in peer-reviewed scientific journals, presented at relevant national and international conferences, and an open-access final report submitted to the funder.

**Trial registration number:**

ISRCTN14409785. Protocol V. 8.0, 14 November 2022.

**STRENGTHS AND LIMITATIONS OF THIS STUDY**

- To date, this is the largest cell therapy randomised trial in children with recessive dystrophic epidermolysis bullosa (RDEB).
- This study incorporates a placebo comparator arm to investigate the efficacy of umbilical cord-derived mesenchymal stem cell use for RDEB, improving the reliability of results.
- The study is double-blinded, with only the pharmacists aware of treatment allocation, eliminating response bias and the placebo effect.
- The study uses a mixed-methods approach, using qualitative analysis to try and collate evidence.
- RDEB is a rare condition, and the sample size of 36 is based on feasibility and not formal power calculations.

## Introduction

 Epidermolysis bullosa (EB) is a heterogeneous group of inherited disorders characterised by mucocutaneous fragility. Recessive dystrophic epidermolysis bullosa (RDEB) is caused by loss of function mutations in the type VII collagen gene (COL7A1), leading to reduced (RDEB intermediate) or absent (RDEB severe) type VII collagen (C7).^1^ This results in slow wound healing and can lead to fibrosis, limb contractures and an increased risk of developing squamous cell carcinoma at sites of chronic wounds and scarring.^2^ Epidemiological data on EB are variable across countries, but the latest epidemiology study from England and Wales estimated a prevalence for RDEB of 3.3 per million population and an incidence of 8.1 per million live births.^3^

Patients with RDEB experience a significant impact on quality of life and severe limitations in function and social activities. Furthermore, the humanistic and economic burden of RDEB extends beyond the patient to affect families and their interpersonal relationships.^4^

In the face of such a significant disease burden, management is currently supportive, involving a multidisciplinary team. Recent years have witnessed a collective global effort in search of effective therapies for RDEB through partnerships between academia, industry, EB charities and patients.^5^ Cell-based therapy encompasses a variety of therapies that include primary keratinocytes, fibroblasts, haematopoietic cells and mesenchymal stem/stromal cells.^6^

The fact that RDEB is considered a systemic inflammatory disease rather than a skin-limited disorder has set the basis for new investigations.^7 8^ Reported clinical trials of cell-based therapies for RDEB comprise intradermal allogeneic fibroblasts,^9 10^ bone marrow transplantation,^11^ mesenchymal stem cells (MSCs)^12^ and intravenous MSCs in children^13^,^16^ and adults with RDEB^15^,^17^ showing promising results.

The overall aim of MissionEB is to assess whether repeated infusions of umbilical cord-derived mesenchymal stem cells (UC-MSCs) are safe and can benefit children with RDEB. The primary objectives are to assess the:

1. Safety of third-party intravenous UC-MSCs in children with RDEB (internal phase I dose de-escalation study for safety gatekeeping).
2. Efficacy of third-party intravenous UC-MSCs in improving disease severity in children with RDEB in the main study (crossover and open-label).

The secondary objectives for the main study are to:

1. Assess the safety of repeated UC-MSCs in children with RDEB.
2. Assess the efficacy of repeated UC-MSCs in improving quality of life and symptoms (eg, pain, itch) in children with RDEB.
3. Undertake a health economic analysis to assess the costs and consequences of treatment with UC-MSCs versus usual care.
4. Explore patients’ and parents’ views in relation to treatment effectiveness and acceptability.

## Methods and analysis

Full detailed methods of the MissionEB trial are described in the full trial protocol, available via the trial registry (https://www.isrctn.com/ISRCTNISRCTN14409785) (online supplemental file 1).

This is a randomised (1:1), placebo-controlled, double-blinded crossover (A/B) trial with an internal phase I dose de-escalation trial in the first 3 months and a 12-month continued treatment follow-on open-label study following review of the data.

The planned overall study start date is 1 August 2020, and the overall study end date is 31 February 2027.

### Internal phase I dose de-escalation trial

**Figure 1:**
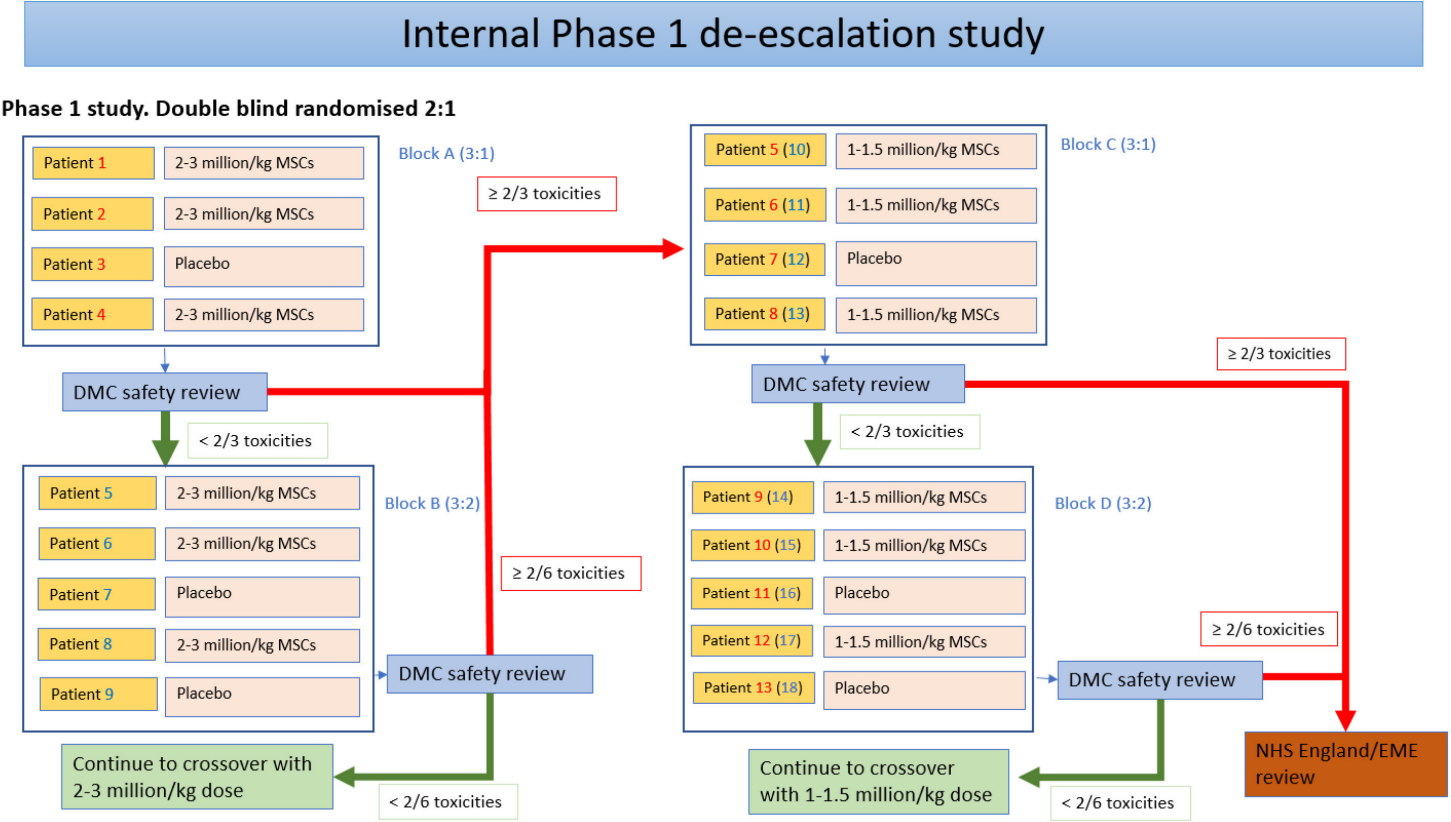
Internal dose de-escalation phase. DMC, data monitoring commtitee; EME, Efficacy and Mechanism Evaluation; MSCs, mesenchymal stem cells; NHS, National Health Service.

An internal phase I study will be conducted on the first nine participants in Great Ormond Street Hospital (GOSH) in two cohorts (4+5 design) as displayed in figure 1. This is only for safety gatekeeping and not to find the optimal dose. Each child will undergo an initial screening, including physical examination, assessment of vital signs and disease severity assessment. Using an overall 2:1 (UC-MSCs:placebo) randomisation ratio, we will recruit four participants (each receiving the full treatment of two infusions before the next participant begins treatment) and randomise them 3:1 (UC-MSCs:placebo). Outcomes will be measured at screening, both infusion visits and then at the 3-month follow-up. Data will be reviewed by the Data Monitoring and Ethics Committee (DMEC). If toxicities, defined as suspected unexpected severe adverse reaction (SUSAR) within 48 hours of infusion, are found in one (or fewer) participant receiving the active treatment, we will proceed to confirm the safety of this dose; a further five participants will be randomised 3:2 (UC-MSCs:placebo). If no further toxicities are found, we will progress to the main two-period crossover study. Of note, dose de-escalation or expansion decisions will be based on three participants randomised to UC-MSCs in each cohort receiving at least one infusion.

### Main crossover trial

**Figure 2:**
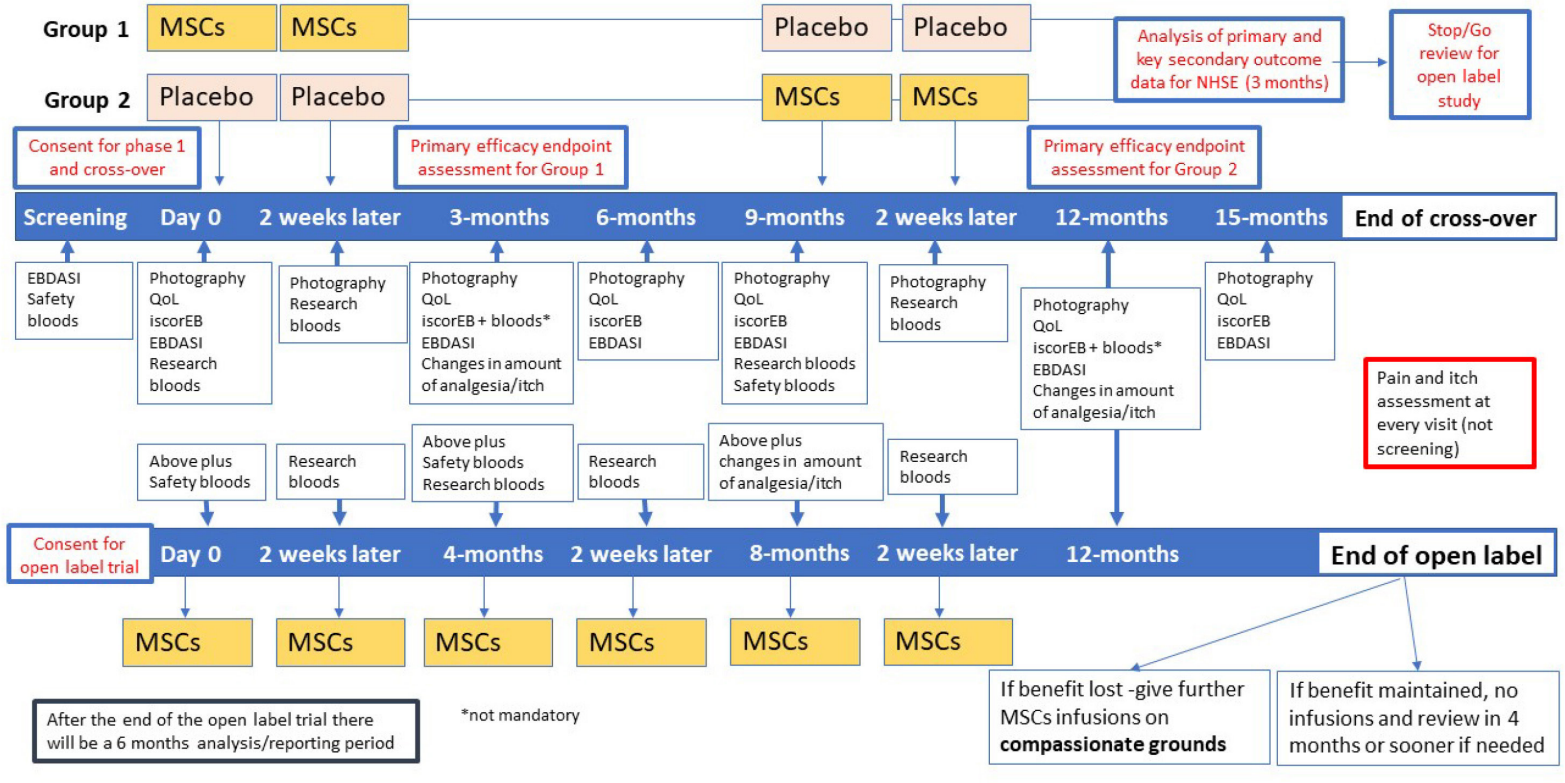
Overview of the phase I, crossover and open-label study timelines. EBDASI, Epidermolysis Bullosa Disease Activity and Scarring Index; MSCs, mesenchymal stem cells; QoL, quality of life.

Each child will undergo an initial screening, including physical examination, assessment of vital signs and disease severity assessment. All study participants will be randomised to receive two consecutive intravenous MSCs or placebo infusions on days 0 and 14 (figure 2). After outcome assessment at 9 months, all children will be crossed over and receive either placebo or UC-MSCs at 9 months and 14 days later. The placebo effect, if any, is expected to tail off by 3 months. In the EBSTEM trial, the maximum benefit of the UC-MSCs was seen at 3 months, and in one patient the beneficial effects lasted for up to 6 months.^14^ This was the primary reason behind the 9-month washout period.

Outcomes will be measured at 0, 3, 6, 9, 12 and 15 months. All children will be followed up every 3 months as part of their clinical care for the first year following the first infusion. A cost consequence analysis will also be undertaken.

We will explore the impact of the treatment on participants by conducting interviews with children and parents (n=10 dyads or individuals) in both arms at the 3-month and 12-month follow-up timepoints.

### Open-label non-randomised study

The open-label study will go ahead if the treatment is found to be effective without safety concerns by the National Institute of Health Research (NIHR) and the National Health Service (NHS) England. MissionEB is not an adequately powered study for feasibility reasons, and as such, the judgement on the efficacy of UC-MSCs will be based on the totality of evidence from all clinical (primary and secondary) outcomes. The criteria for starting the open-label trial are based on the absence of any SUSARs which the DMEC consider to be of clinical concern. In addition, as this is a naturally progressive disease, the study will continue if there is an improvement in any of the primary or secondary outcomes for participants (improvement in disease severity, pain and itch and quality of life). The DMEC and Trial Steering Committee (TSC) will review and consider whether the data indicate evidence of improvement. Participants of the crossover trial will be invited to the open-label study and be given six infusions in total (two infusions at four monthly intervals, at day 0, month 4 and month 8) and followed up at month 12 and outcome measures taken at each visit. No placebo will be administered in the open-label study.

### Recruitment

The trial will be conducted at two sites in England, GOSH and Birmingham Children’s Hospital, which both specialise in paediatric dermatology and are nationally commissioned centres for paediatric EB. Informed consent will be taken by appropriately trained staff as per Section 5.5 of the full protocol.

### Participant eligibility

#### Inclusion criteria

1. Patients who have a diagnosis of RDEB characterised by partial or complete C7 deficiency, including generalised severe and generalised intermediate subtypes.
2. Patients who are over 6 months and before their 16th birthday at time of consent. (Participants must be recruited before their 16th birthday to the crossover trial as this will allow for completion of the whole trial (crossover and open-label) before they are 18 and transition to adult services. However, if there are delays to the study due to dose de-escalation, all participants should be allowed the opportunity to partake in the open-label.)
3. Patients whose responsible parent/guardian has voluntarily signed and dated an Informed Consent Form prior to the first study intervention. Whenever the minor child is able to give consent, the minor’s assent will be obtained in addition to the signed consent of the minor’s legal guardian.

#### Exclusion criteria

1. Patients with other subtypes of EB such as EB simplex, dominant DEB, junctional EB and Kindler EB.
2. Subjects who have received oral or topical corticosteroids for more than seven consecutive days within 30 days of enrolment into this study, with the exception of the following steroids with non-systemic effects and intended to relieve oesophageal symptoms: oral viscous budesonide and inhaled fluticasone.
3. Patients with a known allergy to any of the constituents of the investigational product.
4. Patients with signs of active infection that requires treatment with oral or intravenous antibiotics within 7 days of screening.
5. Patients with a medical history or evidence of active malignancy, including cutaneous squamous cell carcinoma.
6. Patients with both (a) positive C7 ELISA and (b) a positive indirect immunofluorescence with binding to the base of salt split skin at screening.
7. Patients who are pregnant or of childbearing potential who are not abstinent or practising an acceptable means of contraception, as determined by the investigator, for the duration of the treatment phase.
8. Patients having received MSCs from any source in the last 9 months.
9. Simultaneous or previous participation in any interventional trial within 3 months before entering this trial but participation in simultaneous registry and diagnostic trials during the trial is allowed.

### Randomisation and blinding

During both the internal phase I dose de-escalation trial and the main crossover trial, the allocation sequence will be generated by the randomisation statistician using a validated web-based randomisation system within the Sheffield Clinical Trials Research Unit (CTRU). Site research staff blind to the treatment allocation will enter their details on the randomisation system. The randomisation email will be received by the pharmacy and the UC-MSC manufacturer (INmuneBio), who will make up the participant treatment as instructed (either placebo or active treatment) and label this with the participant identifier.

For the internal dose de-escalation trial, participants will be randomised in four cohorts and the choice of the cohort block depends on toxicity decisions made by the DMEC after each cohort (figure 1). The first nine participants are divided into two cohorts, all allocated using simple randomisation: the first four participants will be randomised (3:1) to receive (UC-MSCs:placebo) and the second five participants will be randomised (3:2) to receive (UC-MSCs:placebo). This gives an overall allocation (6:3) for these nine participants to receive (UC-MSCs:placebo). It should be noted that treatments stated here are technically for the first period of the crossover trial. These participants will only receive their second period treatments of the crossover if no concerning toxicity issues are found.

For the main crossover trial, participants will be randomised (to achieve 1:1 overall allocation) to either receive UC-MSCs (in period 1) followed by a placebo (in period 2) or placebo (in period 1) followed by UC-MSCs (in period 2) using simple blocked randomisation. Only the randomisation statistician will have access to the block size during the trial. Participants who have already received period 1 and 2 treatments on the correct UC-MSCs dose during the internal dose de-escalation trial will not be re-randomised again in the main crossover trial as they have contributed valid data for both periods. However, in the event that the dose needs to be de-escalated, participants who previously took part in the dose de-escalation study on a higher dose will washout for at least 9 months and be re-randomised to take part in the main crossover trial using the new, lower dose.

This is a double-blinded study, so all participants and the research team will be unaware of the treatment allocation. Intended unblinding will only occur after the crossover trial during the extended open-label study.

### Intervention and placebo control

#### Internal phase I dose de-escalation trial

The investigational medicinal product (IMP) is a suspension of allogenic UC-MSCs, in a solution containing Dulbecco’s phosphate buffer saline, ZENALB (a solution of human albumin serum) and dimethyl sulfoxide. The placebo consists of the same solution, minus the allogenic UC-MSCs. The full composition of the IMP and placebo is described in Section 6.1 of the full protocol. Each participant will receive two intravenous infusions (days 0 and 14), administered as a slow bolus. At the start, participants within a cohort will receive 2–3×10^6^ cells/kg (or placebo). If dose de-escalation is triggered based on observed toxicity (figure 1), then participants in subsequent cohorts will receive 1–1.5×10^6^ cells/kg (or placebo).

#### Main crossover trial

Each participant will receive a total of four intravenous infusions. Treatment period 1 infusions are administered on days 0 and 14 (either UC-MSC or placebo, depending on the randomised allocation). For the treatment period 2 participants, crossover treatment and the infusions are administered at month 9 and 2 weeks later, administered as a slow bolus over 10 min. The rationale for treatment dosing and washout periods is described in Section 2.2.1 of the full protocol. If it is not necessary to de-escalate the dose following phase I, participants will receive 2–3×10^6^ cells/kg. If a dose de-escalation is required, dosing schedules will be reduced as outlined in figure 1.

#### Open-label study

Each study participant will receive a total of six intravenous infusions (days 0 and 14, month 4 and 2 weeks later, month 8 and 2 weeks later). If it is not necessary to de-escalate the dose following phase I, participants will receive 2–3×10^6^ cells/kg; otherwise, dosing schedules will be reduced as outlined in figure 1.

### Aims and objectives

The overall aim of this study is to assess whether repeated infusions of UC-MSCs are safe and can benefit children with RDEB.

### Primary outcomes

#### Internal phase I dose de-escalation trial

Toxicity as defined by a participant experiencing a SUSAR within 48 hours of a participant receiving an infusion.

#### Main crossover trial

The primary outcome will be change in disease severity as measured by the Epidermolysis Bullosa Disease Activity and Scarring Index (EBDASI) at 3 months post-infusion of UC-MSCs (from day 0, where day 0 is the treatment period baseline).

#### Open-label non-randomised study

The primary outcome will be the same as for the main crossover trial but assessed at 4, 8 and 12 months from day 0 of the open-label study.

MissionEB is not an adequately powered study for feasibility reasons, and as such, the judgement on the efficacy of UC-MSCs will be based on the totality of evidence from all clinical (primary and secondary) outcomes.

### Secondary outcomes

The secondary outcomes applicable for the internal phase I dose de-escalation and crossover parts of the study are:

- Change in EBDASI total score at 6 months post-infusion (from day 0, period baseline).
- Change in disease severity measured by iscorEB at 3 and 6 months post-infusion (from day 0, period baseline).^18^
- Change in general clinical appearance of skin disease as assessed by clinical photography at 3 and 6 months post-infusion (from day 0, period baseline).
- Change in pain and itch as assessed by the Wong–Baker FACES Pain scale for children over 6 years old^19^ and Leuven itch scale scores^20^ at 3 and 6 months post-infusion (from day 0, period baseline).
- Additionally, for pain and itch, changes in the amount of analgesia and itch medications required will be assessed. Participants or their guardians will be asked to detail what pain and itch medication the participant has taken in the last 48 hours, including dose and frequency. At 3 months post-infusion, clinicians blinded to treatment allocation will compare whether this is unchanged, increased or decreased since baseline (day 0, period baseline).
- Change in quality of life according to validated Child Health Utility 9D (CHU-9D) scoring system (16) at 3 and 6 months post-infusion. Quality-of-life assessment will be conducted using CHU-9D. The CHU-9D is a sensitive and validated nine-item child health-related quality-of-life assessment scale developed specifically with and for children and will be used in children aged 7 years and over. An age appropriate by proxy version will be used for children aged 3–6.
- A health economic analysis to assess the costs and consequences of treatment with UC-MSCs versus usual care.
- Adverse events (AEs) and serious adverse events (SAEs) both during the trial and long-term AEs after the trial.
- Safety bloods (routine blood tests and C7 antibodies).
- Research bloods (will be stored for further analysis following a separate research application).
- Serum for cytokines, interleukin (IL)-10, IL-13, IL-22, tumour necrosis factor-alpha at screening, day 0, day 14, month 9 and 2 weeks later in the crossover trial and all visits for the open-label study, except the final 12-month visit.

The outcomes above are also applicable for the open-label for months 4, 8 and 12.

### Safety monitoring and AE reporting

All AEs and adverse reactions will be recorded. SAEs will be subject to expedited reporting requirements. Hospitalisations that are expected to take place as a result of disease progression will not be subject to these reporting requirements. Definitions of AEs and adverse reactions, and a list of exempt events are detailed in Section 9 of the full protocol.

### Trial oversight

The TSC and the DMEC consist of independent members (experts in the field and patient representatives) who will monitor trial data and progress and oversee the trial implementation on behalf of the funder and sponsor. The day-to-day running of the trial is coordinated by the Trial Management Group (TMG), which consists of grant co-applicants and CTRU representatives.

### Sample size

RDEB is a rare condition, and the sample size of 36 (for the crossover trial) is based on feasibility, including availability of the patients and not formal power considerations. As such, the statistical analysis focuses on estimation rather than hypothesis testing. Table 1 gives the standardised widths for the precision of the trial (for a continuous outcome) as assessed by the half-width of a 95% CI.

**Table 1:** Standardised widths for the precision of the trial

Completed	Precision
36	0.49
30	0.53
25	0.59

The sample sizes for safety gatekeeping during the internal dose de-escalation phase are based on a 4+5 design, which is a variant of a 3+3 design with controls to allow seamless transition into the main crossover trial. The open-label follow-on study will depend on available participants for whom further treatment is deemed appropriate following the crossover trial.

### Statistical analysis

#### Internal dose de-escalation phase

The objective of this phase is to monitor the safety of the proposed dose based on the assessment of toxicity data that relates to all SUSARs due to study treatment as deemed by the study clinicians. These data will be assessed by the DMEC, who will recommend whether to continue with the proposed dose, halve the proposed dose to the main crossover trial or stop the trial if the proposed dose is deemed unsafe.

#### Crossover trial

The main crossover trial will be reported according to the Consolidated Standards of Reporting Trials extension for reporting randomised crossover trials.^21^ The primary analysis will be based on the intention-to-treat principle that will include participants with outcome data on two periods of the crossover design.

The study is not formally powered, and estimation rather than formal hypothesis testing is the primary aim of the analysis, although p values may be provided when frequentist statistical models are fitted.

The primary outcome is the change in disease severity as measured by the change in EBDASI total score (across all five domains) at 3 months from day 0.^22^ This outcome will be analysed using a linear mixed-effects model that will include treatment, period and baseline (for each period, if necessary) in the model with a random effect on the participant. The difference in means (mean difference) with 95% CIs giving a range of plausible effects will be estimated using restricted maximum likelihood methods and Satterthwaite df.

To aid interpretation and ability to make probabilistic statements about the distribution of the treatment effect, an equivalent Bayesian linear mixed-effects model will be fitted using non-informative priors on model parameters. This will allow us to estimate the probabilities of the mean difference (treatment effect) being within a certain interval of potential interest to clinicians. For example, the probability of UC-MSCs causing any improvement in disease severity. The analysis of the primary outcome at 3 months and all secondary continuous outcomes will be analysed similarly. In case of missing data, the missing data mechanism will be explored, and multiple imputation may be applied as a sensitivity analysis as appropriate (where necessary).

Other sensitivity analyses will be performed to evaluate the robustness of the primary analyses. The statistical analysis plan will detail methods, including handling of endpoints measured across multiple domains.

There will be no interim analyses during the crossover trial and open-label follow-on study. However, safety data, including toxicities, will be monitored continuously by the DMEC throughout the trial.

It should be noted that judgements on the efficacy of UC-MSCs will be based on the totality of evidence from both primary and secondary clinical outcomes.

#### Open-label follow-on study

We plan to undertake no formal statistical analyses of the 12-month open-label data. This follow-on phase does not have a control group, so no formal comparisons will be made. As a result, the outcomes assessed during the 12-month continued treatment open-label study will be analysed descriptively based on available data. The objective is to assess whether the efficacy observed in the crossover phase (if any) is maintained over these 12 months.

### Qualitative study

We will aim to interview at least 10 participant and parent pairs (approximately five from each site and from both arms) at the 3-month and 12-month visit during the trial to gather their views on their symptoms following treatment and the impact on their lives. This information will add value to the quantitative data and help commissioners to contextualise the findings. We will seek TSC patient and public involvement input in developing the interview questions and methods used in the interviews. The TMG will be involved with the topic guide development, and a research assistant/qualitative researcher will undertake the interviews. Interviews will be conducted by a blinded researcher, with children aged 6 and over, and we will interview parents of all age groups where possible. We will aim to interview the children and parents separately while maintaining comfort for the child and parent.

### Health economics

We do not expect the intervention to lead to any reduction in the cost of care over the study period. This is due to the fact the children will stay on their medication and will continue to have to attend reviews and investigations, and will continue with their current skin care. Even if the skin and wounds improve, the way they will be dressed is unlikely to change as often the dressings are used for protection. As such, we will not collect resource use data on current treatments, and the cost analysis will focus on the costs associated with the infusion of UC-MSCs.

Health benefits will be measured using the CHU-9D, with quality-adjusted life-years compared over the randomised interval.

Mathematical modelling, including external evidence, will be used to explore the potential longer-term health benefits and costs associated with UC-MSCs (eg, long-term reductions in costs of bandages and dressings, avoidance of skin cancer). This analysis will be exploratory.

### Patient and public involvement

Patients and their families were involved in trial design. The Young Persons Advisory Group at GOSH was consulted on document development and meets annually. A patient representative is an independent member of the TSC. They will be involved in the trial results dissemination.

## Ethics and dissemination

Before initiation of the study at participating sites, the protocol, informed consent forms and information materials to be given to the participants will be submitted to an NHS Research Ethics Committee for approval. In addition, the study will be submitted for Health Research Authority (HRA) review and approval. Recruitment of study participants will not commence until the letter of approval has been received from the HRA.

We will endeavour to disseminate the results of the study through peer-reviewed scientific journals and at clinical and academic conferences, as well as submission of a final report to the funder, which will be made available online.

Details of the study will also be made available on the Sheffield CTRU website. Summaries of the research will be updated periodically to inform readers of ongoing progress. The results will be published on a freely accessible database within 1 year of completion of the trial. Trial participants and families will be informed of the trial findings.

### Data management and monitoring

Details of data management and monitoring are described in Sections 14 and 15 of the full trial protocol. This was undertaken in accordance with Sheffield CTRU processes. Participant confidentiality will be respected at all times, and the principles of the General Data Protection Regulation will be followed.

### Protocol amendments

Protocol amendments will require approval of regulatory bodies as per HRA regulations and will be disseminated to relevant parties as per CTRU Standard Operating Procedures. Changes made to the protocol can be found in table 2.

**Table 2:** Changes made to the protocol

Timepoint	Changes made
Set-up	
2 June 2021	Blinding process clarified. Process for assessing use of analgesia updated to every visit alongside concomitant medications. Infusion procedure clarified to slow bolus administration and addition of paracetamol as supportive medication for all patients. Leuven itch to be completed by patients over 14 years of age. Age-appropriate proxy version of CHU-9D for children 3–6 years old. Clarification that long-term safety data collection in the event of patient withdrawal from follow-up will be collected unless a patient withdraws consent.
23 September 2021	Update to the presentation of IMP from 2 mL cryovials containing 1 × 10^7^ CORDStrom (concentration of 0.5 × 10^7^ /mL), to 50 mL CryoMACS bags with a fill volume of 10 mL or 15 mL cell suspension per bag and a minimum concentration of 3 × 10^6^ CORDStrom per mL. Clarification that a clinician will assess changes to pain and itch medication using information about what medication the participant has taken in the last 48 hours prior to the study visits. Update that pharmacists will be unblinded to treatment allocation to complete QP checks
Recruitment	
7 March 2022	Addition of the outcome measure ‘change in EBDASI’ at 6 months post-infusion. Exclusion criteria 2 amended to clarify that it only excludes those that have been taking corticosteroids for more than seven consecutive days. Use of budesonide is exempted as low-dose budesonide is used in EB to alleviate oesophageal strictures and does not have a systemic anti-inflammatory effect. Several minor corrections and clarifications around how/when the outcome measures will be collected. The purposes of these changes are to clarify the procedures.
19 August 2022	Exclusion criteria 2 amended to allow for patients who are on inhaled fluticasone to be included in the study. Routine bloods have been added at visits 4 and 8 and 15. If clinical blood results are not available between baseline and the visit, a blood test can be taken at month 3 follow-ups, but it is not mandated. Information about the qualitative research has been added.
Intervention	
5 April 2023	Increase in allowed windows for dosing. Clarification of calculating follow-up visits.

CHU-9D, Child Health Utility 9D; EB, epidermolysis bullosa; EBDASI, Epidermolysis Bullosa Disease Activity and Scarring Index; IMP, investigational medicinal product; QP, qualified person.

## Supplementary material

10.1136/bmjopen-2024-089857 online supplemental file 1
