# Supplementary material for: Mesenchymal intravenous stromal cell infusions in children with recessive dystrophic epidermolysis bullosa: MissionEB protocol for a randomised, double-blinded, placebo-controlled, two-centre, crossover trial with an internal phase I dose de-escalation phase and open-label extension
Source: BMJ Open. 2025 May 21;15(5):e089857. doi: 10.1136/bmjopen-2024-089857 (PMC12097048; doi:10.1136/bmjopen-2024-089857)
Supplement: online supplemental file 1 [file bmjopen-15-5-s001.docx]

| Data category | Information |
| --- | --- |
| Primary registry and trial identifying number | ISRCTN  ISRCTN14409785 |
| Date of registration in primary registry | 25/03/2021 |
| Secondary identifying numbers | EuDRACT 2020-005049-18 |
| Source(s) of monetary or material support | This project is funded by the National Research Collaboration Programme (NIHR127963), an NHS England and National Institute for Health Research partnership. Additional funds are also provided by CureEB. |
| Primary sponsor | Greta Ormond Street Hospital for Children NHS Foundation Trust |
| Contact for public queries | missioneb@sheffield.ac.uk |
| Contact for scientific queries | [anna.martinez@gosh.nhs.uk](mailto:anna.martinez@gosh.nhs.uk) |
| Public title | Mesenchymal stromal cell therapy for children with recessive dystrophic epidermolysis bullosa |
| Scientific title | Double-blinded placebo-controlled crossover study of Mesenchymal Intravenous Stromal cell Infusions in children with recessive dystrophic Epidermolysis Bullosa |
| Countries of recruitment | England |
| Health condition(s) or problem(s) studied | Recessive Dystrophic Epidermolysis Bullosa (RDEB) |
| Intervention(s) | Umbilical Cord derived Mesenchymal Stromal Cells (CORDStrom) |
|  | Placebo |
| Key inclusion and exclusion criteria | **Inclusion**  1. Diagnosis of RDEB characterised by partial or complete C7 deficiency including generalised severe and generalised intermediate subtypes  2. Aged >6 months and <16 years at time of enrolment  3. Responsible parent/guardian has voluntarily signed and dated an Informed Consent Form (ICF) prior to the first study intervention. Whenever the minor child is able to give consent, the minor’s assent will be obtained in addition to the signed consent of the minor’s legal guardian. |
|  | **Exclusion**  1. Diagnosis of RDEB characterised by partial or complete C7 deficiency including generalised severe and generalised intermediate subtypes  2. Aged >6 months and <16 years at time of enrolment  3. Responsible parent/guardian has voluntarily signed and dated an Informed Consent Form (ICF) prior to the first study intervention. Whenever the minor child is able to give consent, the minor’s assent will be obtained in addition to the signed consent of the minor’s legal guardian. |
|  |  |
| Study type | Interventional |
|  | Randomised crossover trial |
|  | Prospective double-blind randomized placebo-controlled cross-over trial incorporating a phase 1 de-escalation study and a possible continued treatment follow-on open-label study |
|  |  |
| Date of first enrolment | 06/10/2021 |
| Target sample size | 36 |
| Recruitment status | Complete |
| Primary outcome(s) | Disease severity measured using the total score across all 5 domains of the Epidermolysis Bullosa Disease Activity and Scarring Index (EBDASI) at day 0 and 3 months post-infusion of UC-MSCs |
| Key secondary outcomes | Safety measured using monitoring of AEs and SAEs throughout the trial.  Change in quality of life according to validated Child Health Utility 9D (CHUD-9D) scoring system  Change in disease severity measured using instrument for scoring clinical outcomes of research for epidermolysis bullosa (iscorEB) at 3- and 6-months post infusion  Change in general clinical appearance of skin disease measured using clinical photography at 3- and 6-months post infusion  Change to pain and itch as assessed by the Wong-Baker FACES Pain scale for children over 6 years old and Leuven itch scale scores at 3- and 6-months post infusion  Change to pain and itch as assessed by the amount of analgesia and itch medications required  Health economic analysis to assess the costs and consequences of treatment with UC-MSCs versus usual care |
