# Supplementary material for: Mesenchymal intravenous stromal cell infusions in children with recessive dystrophic epidermolysis bullosa: MissionEB protocol for a randomised, double-blinded, placebo-controlled, two-centre, crossover trial with an internal phase I dose de-escalation phase and open-label extension
Source: BMJ Open. 2025 May 21;15(5):e089857. doi: 10.1136/bmjopen-2024-089857 (PMC12097048; doi:10.1136/bmjopen-2024-089857)
Supplement: Supplementary file 2 [file bmjopen-2024-089857.reviewer_comments.pdf]

PEER REVIEW HISTORY

BMJ Open publishes all reviews undertaken for accepted manuscripts. Reviewers are asked to complete a checklist review form and are provided with free text boxes to elaborate on their assessment. These free text comments are reproduced below.

ARTICLE DETAILS

Title (Provisional)

Mesenchymal intravenous stromal cell infusions in children with Recessive Dystrophic Epidermolysis Bullosa: MissionEB protocol for a randomised, double-blinded, placebo controlled, two centre, cross-over trial with an internal phase 1 dose de-escalation phase and open label extension.

Authors

Bageta, Maria; López-Balboa, Pablo; Dimairo, Munyaradzi; Glover, Rachel; Hutchence, Kate; Papaioannou, Diana; Cooper, Cindy; Biggs, Katie; Tappenden, Paul; Ennis, Katherine; Ogboli, Malobi; Lovgren, Marie-Louise; Poudel, Pratima; Nadeem, Muna; McGrath, John A; Julious, Steven A; Petrof, Gabriela; Martinez, Anna

---

VERSION 1 - REVIEW

**Reviewer**	**1**
**Name**	**Odorisio, Teresa**
**Affiliation**	**Istituto Dermopatico dell'Immacolata IRCCS**
**Date**	**12-Jul-2024**
**COI**	**None**

This is a phase I/II clinical trial in paediatric patients with a rare skin disease for which there is an unmet therapeutic need. The study has a rather complex design aimed at maximising results with an inevitably limited number of patients, including a crossover study. It is innovative for the reasons cited by the authors: the largest cohort of paediatric patients to date in a cell-based trial for this rare disease, the double-arm and double-blind design. Importantly for a systemic disease such as recessive dystrophic epidermolysis bullosa, umbilical cord mesenchymal stromal cells are administered by intravenous infusion. Stromal cells have been shown to be preferentially delivered to sites of injury, so a systemic effect is likely.

I think this is a very important, well-designed study that takes into account all the variables and possible outcomes. It is well written in all respects.

Please consider the following points.

1. It is not clear to me why the placebo does not contain one of the excipients, Zenalb 4.5 (HAS).

2. The last sentence on page 3 is incomplete.

**Reviewer**	**2**
**Name**	**Calvo, Margarita**
**Affiliation**	**Pontificia Universidad Católica de Chile**
**Date**	**27-Dec-2024**
**COI**	**None**

The protocol is adequate and should be able to answer important questions. My only suggestion is to add questionnaires in pain. For instance, I would include the DN4 which is a screening tool for neuropathic pain, and the a body map like the one included in the FSQ (to localise where is the pain and the itch)

VERSION 1 - AUTHOR RESPONSE

Reviewer 1

Dr. Teresa Odorisio, Istituto Dermopatico dell'Immacolata IRCCS
Comments to the Author:
This is a phase I/II clinical trial in paediatric patients with a rare skin disease for which there is an unmet therapeutic need. The study has a rather complex design aimed at maximising results with an inevitably limited number of patients, including a crossover study. It is innovative for the reasons cited by the authors: the largest cohort of paediatric patients to date in a cell-based trial for this rare disease, the double-arm and double-blind design. Importantly for a systemic disease such as recessive dystrophic epidermolysis bullosa, umbilical cord mesenchymal stromal cells are administered by intravenous infusion. Stromal cells have been shown to be preferentially delivered to sites of injury, so a systemic effect is likely.
I think this is a very important, well-designed study that takes into account all the variables and possible outcomes. It is well written in all respects.
Please consider the following points.

1- It is not clear to me why the placebo does not contain one of the excipients, Zenalb 4.5 (HAS).

Thank you so much for your kind comments. In response to your question, the IMPD lists HAS as a component of the placebo, however this seems to have been omitted from the protocol. We have clarified this in the paper and we will add to the upcoming substantial amendment of the protocol.

2- The last sentence on page 3 is incomplete.

This has been amended.

Reviewer 2

Dr. Margarita Calvo, Pontificia Universidad Católica de Chile
Comments to the Author:
The protocol is adequate and should be able to answer important questions. My only suggestion is to add questionnaires in pain. For instance, I would include the DN4 which is a screening tool for neuropathic pain, and the a body map like the one included in the FSQ (to localise where is the pain and the itch)

Thank you so much for your thoughtful comment regarding the assessment of pain and itch, which are indeed significant comorbidities and challenging to measure accurately in young children, especially when considering both chronic and acute components. To address these challenges in Mission EB, we used age appropriate tools such as Wong-Baker FACES pain score for children over six years old and a visual analogue scale completed by carers/guardians. Additionally, we used the iscorEB patient sub-score, which includes a dedicated section for pain and itch. This domain captures detailed information on pain, including its sources (skin, mouth, eye, bone/join) and overall intensity. For itch, we used the Leuven itch scale for patients over 14 years of age, which provides insights into itch frequency, severity, sensory characteristics. location, and associated circumstances. For younger children, between 4 and 13 years of age, the Itch man scale provides information on severity and interference with activities.

We acknowledge that older children and adults may experience neuropathic pain, which is an important aspect to consider in future studies. Your suggestion is very valuable and we will incorporate it in subsequent research to improve our assessments.

Additional statements:

The authors wish to explain that at the time of article submission the trial was in the data collection stage and statistical analysis had not begun. The word limit has been exceeded in order to respond fully to reviewer comments.
